# Differential protein expression during growth on model and commercial mixtures of naphthenic acids in *Pseudomonas fluorescens* Pf‐5

**DOI:** 10.1002/mbo3.1196

**Published:** 2021-07-19

**Authors:** Boyd A. McKew, Richard Johnson, Lindsay Clothier, Karl Skeels, Matthew S. Ross, Metodi Metodiev, Max Frenzel, Lisa M. Gieg, Jonathan W. Martin, Michael A. Hough, Corinne Whitby

**Affiliations:** ^1^ School of Life Sciences University of Essex Colchester UK; ^2^ Canada's Oil Sands Innovation Alliance Calgary AB Canada; ^3^ Department of Biological Sciences University of Calgary Calgary AB Canada; ^4^ Department of Physical Sciences MacEwan University Edmonton AB Canada; ^5^ Oil Plus Ltd Newbury UK; ^6^ Department of Environmental Science Stockholm University Stockholm Sweden

**Keywords:** naphthenic acids, oil sands process‐affected water, proteomics, *Pseudomonas fluorescens*, tailing ponds, toxicity

## Abstract

Naphthenic acids (NAs) are carboxylic acids with the formula (C*_n_* H*_2n_*_+_ *_Z_* O_2_) and are among the most toxic, persistent constituents of oil sands process‐affected waters (OSPW), produced during oil sands extraction. Currently, the proteins and mechanisms involved in NA biodegradation are unknown. Using LC‐MS/MS shotgun proteomics, we identified proteins overexpressed during the growth of *Pseudomonas fluorescens* Pf‐5 on a model NA (4′‐*n*‐butylphenyl)‐4‐butanoic acid (*n*‐BPBA) and commercial NA mixture (Acros). By day 11, >95% of *n*‐BPBA was degraded. With Acros, a 17% reduction in intensity occurred with 10–18 carbon compounds of the *Z* family −2 to −14 (major NA species in this mixture). A total of 554 proteins (*n*‐BPBA) and 631 proteins (Acros) were overexpressed during growth on NAs, including several transporters (e.g., ABC transporters), suggesting a cellular protective response from NA toxicity. Several proteins associated with fatty acid, lipid, and amino acid metabolism were also overexpressed, including acyl‐CoA dehydrogenase and acyl‐CoA thioesterase II, which catalyze part of the fatty acid beta‐oxidation pathway. Indeed, multiple enzymes involved in the fatty acid oxidation pathway were upregulated. Given the presumed structural similarity between alkyl‐carboxylic acid side chains and fatty acids, we postulate that *P*. *fluorescens* Pf‐5 was using existing fatty acid catabolic pathways (among others) during NA degradation.

## Graphical abstract

Oil sands mining operations produce vast amounts of oil sands process‐affected waters (OSPW) that contain recalcitrant and toxic naphthenic acids (NAs), yet little is known about the mechanisms of NA biodegradation and the enzymes involved. Here, we identified several proteins overexpressed in *Pseudomonas fluorescens* Pf‐5 during growth on NAs and included multiple transporters (e.g. ABC transporters), suggesting a cellular protective response from NA toxicity, alongside proteins associated with the fatty acid, lipid, and amino acid metabolism. We postulate that *P. fluorescens* Pf‐5 is using existing fatty acid catabolic pathways (among others) during NA degradation and these protein targets could be exploited in future biotechnological efforts for OSPW reclamation.

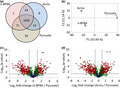

## 1 INTRODUCTION

In the Athabasca region of Northern Alberta, Canada, surface mining and caustic hot water extraction of bitumen have resulted in the accumulation of vast quantities of wastewaters known as oil sands process‐affected water (OSPW) (Quagraine et al., 2005; Siddique et al., 2011). There are still no approved strategies to treat and safely release OSPW; thus, it is contained in large tailings ponds which cause considerable environmental concern (Giesy et al., 2010). One of the major challenges for reclaiming these ponds is the presence of naphthenic acids (NAs) which are the main toxic components of OSPW and demonstrate both acute and chronic toxicity to a variety of aquatic organisms (Beddow et al., 2016; Frank et al., 2009; Headley & McMartin, 2004; Morandi et al., 2015). NAs are also highly persistent under field conditions and a major long‐term strategy for remediation is to age the water in end‐pit lakes, but this strategy is highly uncertain and may take decades (Gosselin et al., 2010). For effective OSPW remediation and site reclamation, effective modes of NA degradation or removal will be crucial.

Despite their recalcitrance and toxicity, very little is known about the mechanisms of NA biodegradation and the enzymes involved (Whitby, 2010). A few studies using model NAs have shown that several microorganisms (e.g., *Pseudomonas putida*) can metabolize single‐ringed NAs by the beta‐oxidation pathway (Clothier & Gieg, 2016; Johnson et al., 2013; Smith et al., 2008). In addition, a *Mycobacterium* sp. was found to degrade aromatic NAs by both the beta‐ and omega‐oxidation pathways (Johnson et al., 2012). Although such studies have made some headway, the proteins involved in NA biodegradation (e.g., transport and metabolic pathways) are still unknown. Their identification has in part been hampered by the chemical complexity of environmental NAs and the identification of the individual components.

It is known that NAs are a complex class of aliphatic, cycloaliphatic, and aromatic monocarboxylic acids (C*_n_* H*_2n_*_+_ *_Z_* O_2_), where *n* is the number of carbon atoms and *Z* is either zero or a negative even integer representing hydrogen deficiency due to double bonds or rings (Brient et al., 1995; Clemente & Fedorak, 2005; Whitby, 2010). However, only a few NA structures have been identified in OSPW, including tri‐, tetra‐, or pentacyclic monocarboxylic acids and monoaromatic species (Rowland et al., 2011a,2011b, 2012; Wang et al., 2013; West et al., 2013). Although aromatic alkanoic acids make up a comparatively small proportion of NA mixtures (<10% in crude oils; Hsu et al., 2000), they are significant contributors to the overall toxicity and recalcitrance of NAs in OSPW (Headley & McMartin, 2004), including notably acting as environmental androgen receptor antagonists (Thomas et al., 2009).

Previous studies have shown that microbial biodegradation decreases OSPW toxicity over time (Frank et al., 2009; Johnson et al., 2011; Quagraine et al., 2005) and the use of microorganisms is a potential remediation strategy for oil sands operators. However, NA biodegradation is affected by chemical structure and the more recalcitrant NAs contain multiple branched alkyl chains and methyl substitution of the cycloalkane rings (Johnson et al., 2012; Smith et al., 2008), as well as the highly branched and multi‐ringed diamondoid NAs, found in tailings ponds (Ahad et al., 2018; Demeter et al., 2015; Folwell et al., 2020; Paulssen & Gieg, 2019). Despite their recalcitrance, several NA‐degrading communities and species have been identified including *Pseudomonas putida* and *Pseudomonas fluorescens* (Del Rio et al., 2006; Johnson et al., 2013). Using *P*. *fluorescens* Pf‐5 as a model organism, this study aimed to identify proteins differentially expressed during growth on a model NA and commercial NA mixture. Identification of such overexpressed proteins could then be targeted as a novel approach for improving OSPW reclamation in the future.

## 2 EXPERIMENTAL PROCEDURES

### 2.1 Naphthenic acids used in this study

**FIGURE 1:**
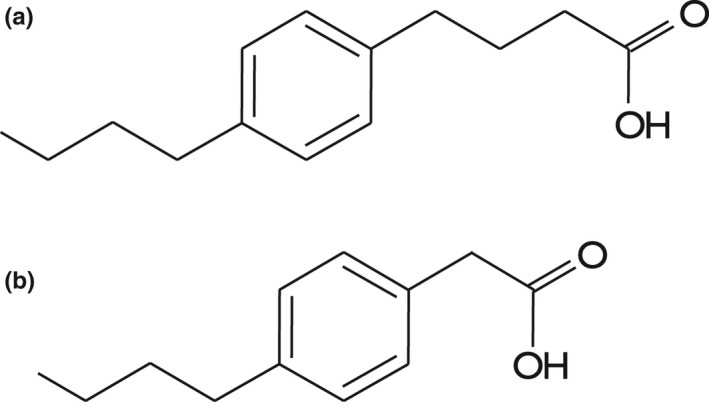
Structure of (4′‐*n*‐butylphenyl)‐4‐butanoic acid (*n*‐BPBA) (a) and (4′‐*n*‐butylphenyl)ethanoic acid (*n*‐BPEA) (b)

The (4′‐*n*‐butylphenyl)‐4‐butanoic acid (*n*‐BPBA) used in this study (Figure 1A) was synthesized using a modified Haworth synthesis (Smith et al., 2008). The commercial mixture of NAs was obtained from Acros Organics, UK (EINECS 215–662–8).

### 2.2 *Pseudomonas fluorescens* Pf‐5 culture

*Pseudomonas fluorescens* Pf‐5 was obtained from Dr. Andrew Spiers (University of Abertay, Aberdeen, UK) and maintained on Luria Bertani (LB) agar (Sambrook et al., 1989). Cultures of *P*. *fluorescens* Pf‐5 were grown overnight at 30°C and 110 rpm in LB broth to an optical density OD_600_ between 0.6 and 0.7. The inoculum was centrifuged at 3435 *g* for 15 min (Heraeus Multifuge 3‐SR) and cell pellets were washed three times with sterile minimal salts medium (MSM) to remove any trace carbon carried over from the LB medium (Johnson et al., 2011). Cells were inoculated (1% (v/v)) into triplicate 100 ml MSM in 120 ml sterile serum bottles containing either *n*‐BPBA (final concentration of 10 mg/l), commercial NA mixture (Acros Organics, UK) (final concentration 100 mg/l) or 1% (w/v) sodium pyruvate. All serum bottles were capped with a PTFE‐lined crimp‐sealed septum. Killed controls were also prepared by addition of HgCl_2_ (2% (w/v) final concentration) to the inoculum before inoculation. Viability was checked by overnight growth on LB agar at 30°C. Abiotic controls were also prepared as well as procedural blanks containing *P*. *fluorescens* Pf‐5 (1% v/v) but no NAs. All bottles were incubated at 110 rpm in the dark at 30°C and OD_600_ was measured spectrophotometrically to monitor growth. Destructive sampling of triplicate bottles was carried out whereby cultures were centrifuged at 9466 *g* for 10 min (Heraeus Multifuge 3‐SR). This was performed for all cultures at day zero and during the early exponential growth phase which was approximately day 11 (for cultures grown on *n*‐BPBA and the commercial NA mixture) and 14 h (for cultures grown on pyruvate). Proteins from cell pellets were extracted immediately and supernatants were frozen at −20°C before ethyl acetate extraction of NAs.

### 2.3 NA extraction and analysis

NAs were extracted from the supernatants using ethyl acetate as previously described (Johnson et al., 2011; Smith et al., 2008). *n*‐BPBA extracts were analyzed by gas chromatography–mass spectrometry (GC‐MS) using an Agilent 7890 GC as previously described (Johnson et al., 2011). For experiments with the commercial NAs, extracts were reduced to dryness under a gentle stream of nitrogen, spiked with 400 ng of ^13^C‐myristic acid (as an internal standard), and reconstituted in 1 ml of 10 mM ammonium acetate in 75:25 H_2_O/acetonitrile (HPLC grade, Fisher Scientific). Samples were analyzed on a Shimadzu LC 20XR LC system using a Waters BET Phenyl (15 cm × 1.0 mm × 1.6 µm d.p., Waters). Ammonium acetate (10 mM, MS grade, Sigma‐Aldrich) in Milli‐Q water (Solvent A) and 10 mM ammonium acetate dissolved in 60:40 methanol/acetonitrile (Solvent B) were used as eluents. The mobile phase composition was held at 5% B for 2 min, followed by a linear gradient to 99% B in 16 min, followed by an isocratic hold at 99% B for 2 min. The solvent composition was returned to 5% B and remained at this composition for 10 min before the next injection. The flow rate was 100 µl/min. An API 5600 (AB Sciex, Framingham, MA) time of flight high‐resolution mass spectrometer with an electrospray source operating in negative ionization mode was used for detection. The acquisition was performed in scan mode from *m/z* 100 to *m/z* 650. Data were acquired using Analyst TF and chromatographic peaks were integrated with Multiquant 2.0. software (AB Sciex, Framingham, MA) as previously described (Ross et al., 2012). All peak areas were normalized to the peak area of the internal standard. The *Z* = 0 family was removed from the data set, as these species have been identified as saturated fatty acids (Ross et al., 2012). The species C10 *Z* = −8 was also removed from the LC data set as this corresponded to the 4‐phenylbutanoic acid added as an internal standard.

### 2.4 Protein extraction and quantification

Cell pellets were resuspended in four volumes of sodium dodecyl sulfate (SDS) sample buffer [comprising 62.5 mM Tris‐HCl pH 6.8, 10% (v/v) glycerol, 2% (w/v) SDS, 12 mM dithiothreitol, and one Pierce Protease Inhibitor Tablet per 50 ml] and boiled for 10 min. The cell debris was removed by centrifugation at 11,357 *g* for 5 min and the supernatant was transferred to a sterile microcentrifuge tube and stored at −80°C.

### 2.5 In‐gel digestion with trypsin

A procedure that is optimized for the digestion of whole‐cell lysates was used as previously described (Metodiev, 2011). The protein samples containing 20 mg total protein in SDS PAGE buffer were loaded onto standard Laemmli‐type polyacrylamide gels and allowed to stack and enter the resolving gel but not to separate. The protein bands were excised and digested with trypsin as described in Alldridge et al. (2008). The peptides were extracted and dried in a vacuum concentrator and reconstituted in 20 μl of LC/MS‐grade water containing 0.1% (v/v) formic acid. The peptide concentration was measured by spectrophotometry using a NanoDrop spectrophotometer and 1 ml aliquot of the reconstituted peptide sample.

### 2.6 LC‐MS/MS analysis of peptides

Peptide analysis was performed as described by Greenwood et al. (2012). Briefly, 2 μg total peptides per sample were injected automatically from the microplate, desalted online, separated on a 15 cm long pulled‐tip nanocolumn, and analyzed by electrospray‐ionization tandem mass spectrometry on a hybrid high‐resolution LTQ/Orbitrap Velos instrument (Thermo Scientific). The raw data files were converted to mzXML format using the ReAdW program and uploaded onto the LabKey server for analysis. The open‐source search engine X! Tandem was used to identify the proteins and acquire spectral count data (Craig & Beavis, 2004). The primary statistical evaluation and filtering of the protein identification and spectral count data were performed as described in Alldridge et al. (2008) using the peptide and protein prophet programs (Nesvizhskii et al., 2003) integrated into the LabKey CPAS (Computational Proteomics Analysis System version 2.2). Peptides and proteins were filtered at a 0.3% false discovery rate (FDR) to obtain the final dataset. Proteins were quantified by counting the number of MS/MS spectra matched to corresponding proteins. Sequences from UniProt for *P*. *fluorescens* (strain ATCC BAA‐477 / NRRL B‐23932 / Pf‐5) were used to perform protein identification. Spectral counts were normalized to the run yielding the highest number of spectral counts (17660) by the TSpC Total Spectral Counts method (Dong et al., 2007) to account for small observed differences between runs (spectral counts ranged from 14,580 to 17,660 per run).

### 2.7 Bioinformatics analysis

Differential expression analysis was performed on 1261 proteins by analysis of variance (ANOVA) and Tukey's HSD test with Benjamini–Hochberg post hoc corrections (Benjamini & Hochberg, 1995) within the XLSTAT Premium Version 2016.1 (Addinsoft) “OMICs” package. All p‐values presented throughout are the modified *p*‐values following Benjamini–Hochberg post hoc correction. Proteins with mean spectral counts less than 3 in any one treatment were excluded from this statistical analysis. Further analysis of individual proteins that demonstrated a significant increase in abundance compared with controls was carried out using NCBI’s blastp program (Altschul et al., 1990; McGinnis & Madden, 2004) and Conserved Domains Database (CDD) (Marchler‐Bauer et al., 2015). Pathway analysis was carried out by examination of Kyoto Encyclopedia of Genes and Genomes (KEGG; https://www.genome.jp/kegg) pathways for *P*. *fluorescens* Pf‐5. Protein enzyme commission numbers were obtained from the identification described above and relevant pathways were examined for the presence of upregulated proteins.

## 3 RESULTS AND DISCUSSION

### 3.1 Growth of *P. fluorescens* Pf‐5 and NA degradation

**FIGURE 2:**
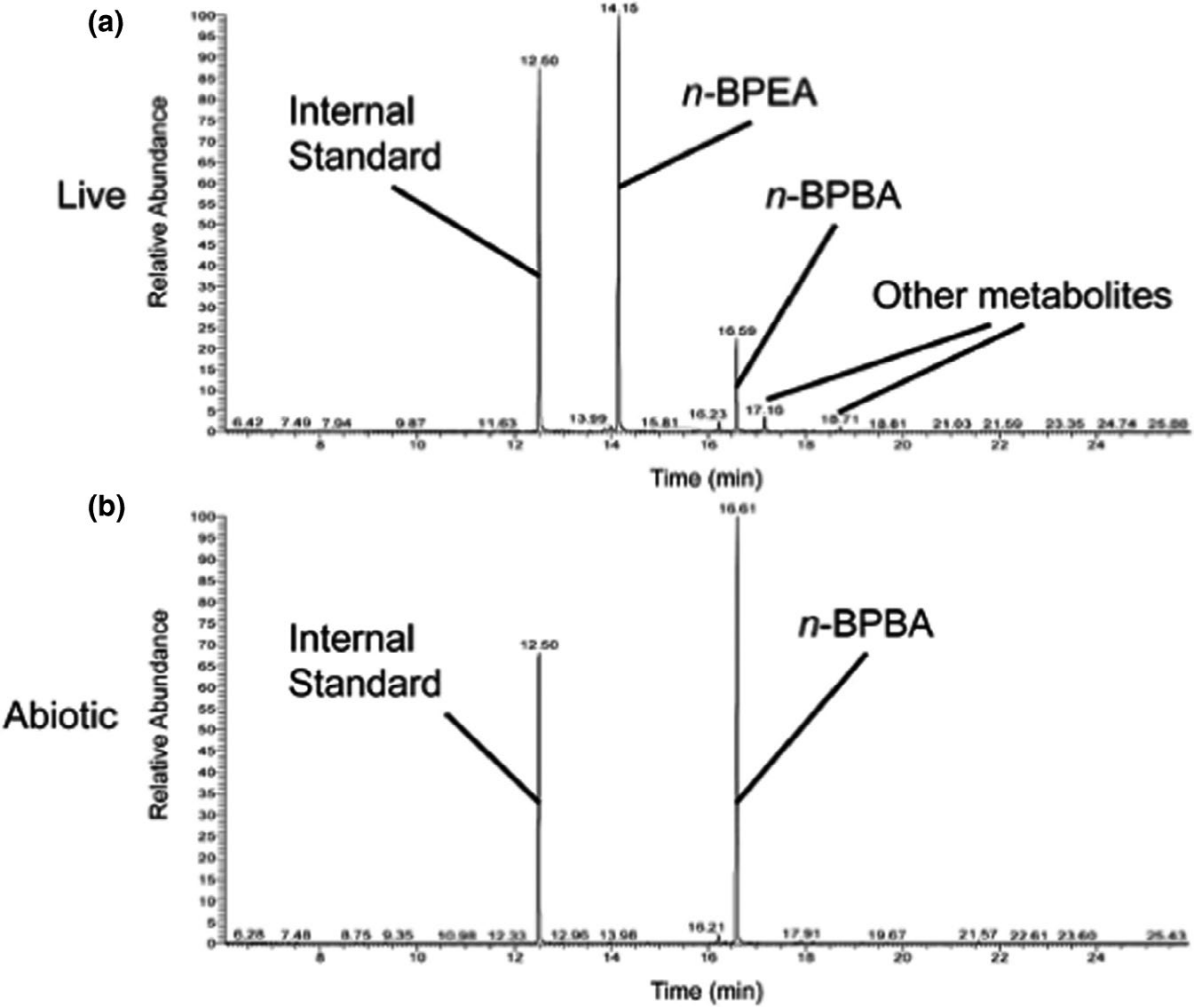
Degradation of *n*‐BPBA and production of (4′‐*n*‐butylphenyl)ethanoic acid (*n*‐BPEA) metabolite by *Pseudomonas fluorescens* Pf‐5 (a) compared to abiotic controls (b) following 11 days of incubation

**FIGURE A1:**
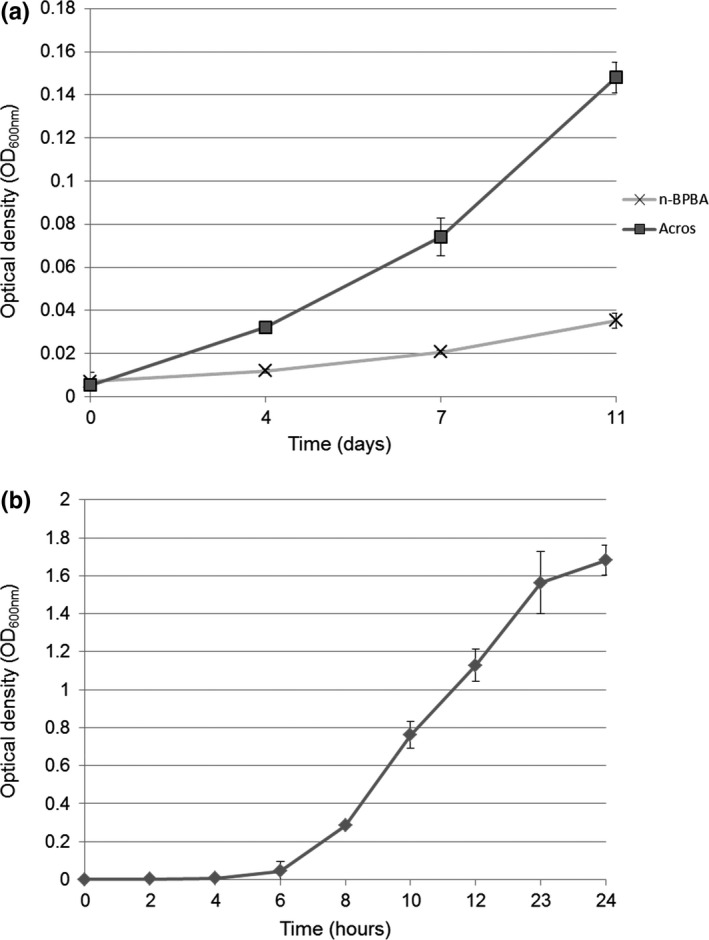
Growth of *Pseudomonas fluorescens* Pf‐5 in minimal salts medium supplemented with either *n*‐BPBA or Acros commercial NA mixture (a) or pyruvate control (b)

*Pseudomonas fluorescens* Pf‐5 grew on all NAs tested with no growth observed in the abiotic or killed controls. After a short lag phase, growth was observed after 6 h on pyruvate and after 4 days on *n*‐BPBA and the commercial NA mixture (Acros) (Figure A1), which is similar to previous NA biodegradation studies (Johnson et al., 2011, 2013). Degradation of *n*‐BPBA occurred by day 11 as shown by a reduced signal in *n*‐BPBA (retention time 16.59 min) compared with the abiotic control (Figure 2). During *n*‐BPBA degradation, a metabolite (4′‐*n*‐butylphenyl)ethanoic acid (*n*‐BPEA) was produced (Figure 1B) which was absent in the abiotic control. This *n*‐BPEA metabolite has been found previously with mixed enrichment cultures (Johnson et al., 2011); and pure cultures of a *Mycobacterium* sp. (Johnson et al., 2012); and *Pseudomonas putida* KT2440 (Johnson et al., 2013). Additional unidentified peaks (with *m/z* between 311–341) were also observed that had longer retention times, suggesting they are less polar or less water‐soluble compounds. These findings confirmed that *P*. *fluorescens* Pf‐5 degraded *n*‐BPBA and that the initial degradation steps involved the removal of two carbons from the carboxyl side chain indicative of beta‐oxidation as previously shown (Johnson et al., 2011, 2012, 2013). In total, <5% of the *n*‐BPBA was remaining after 11 days of incubation. Previous NA biodegradation studies have also shown that *P*. *fluorescens* and *P*. *putida* can degrade model NAs such as cyclohexane carboxylic acids (Blakley & Papish, 1982; Del Rio et al., 2006; Johnson et al., 2013). Pseudomonads also increased in abundance during the degradation of the highly branched aromatic NA (*sec*‐BPBA) by mixed enrichment cultures (Johnson et al., 2011).

**FIGURE 3:**
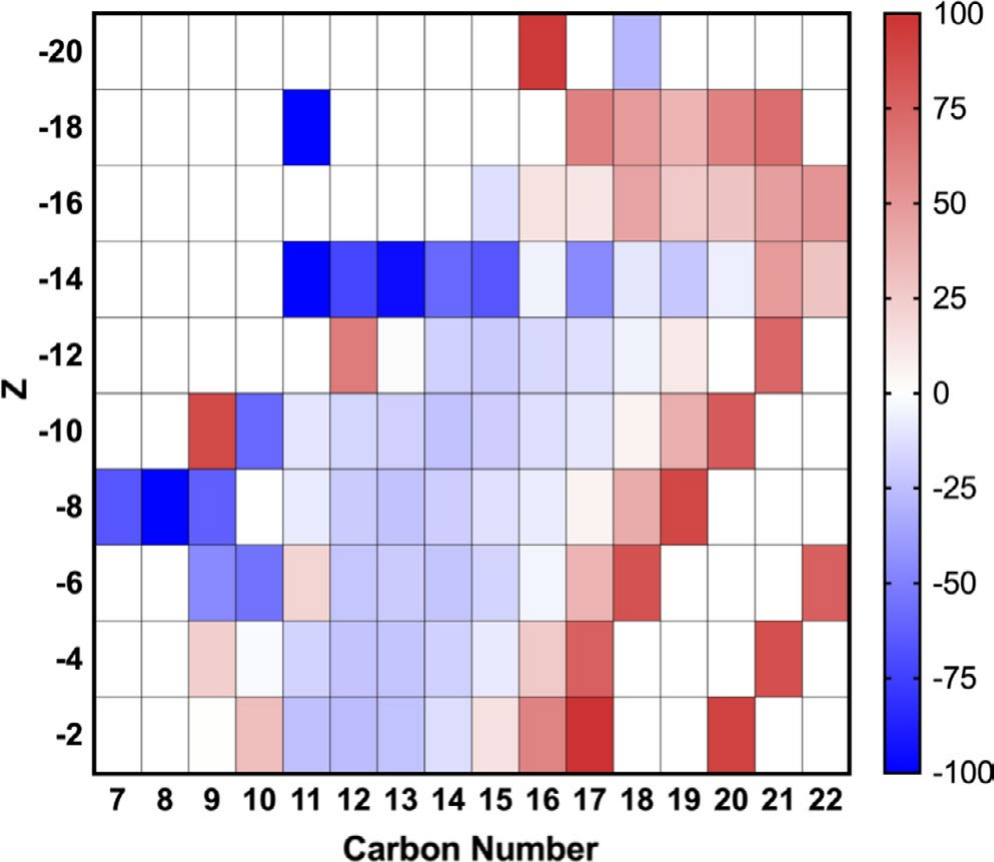
Heat map of the percentage change for Acros commercial NA species, plotted by carbon number and Z, following 11 days of incubation. Percent changes are calculated relative to killed control following 11 days of incubation

**FIGURE A2:**
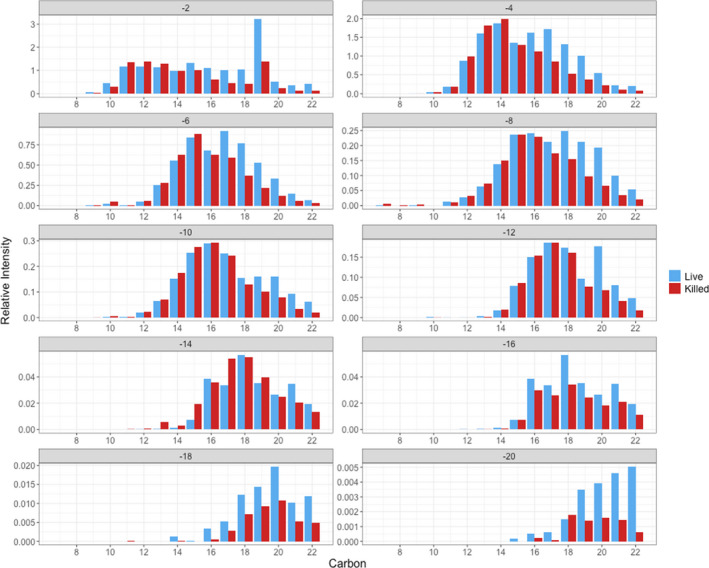
Distribution of NA compounds fitting the molecular formula C_n_H_2n+z_O_2_ from C_7_ to C_22_ faceted by hydrogen deficiency (Z). NAs were acid‐extracted from Pseudomonas fluorescens Pf‐5 cultures containing a commercial NA mixture (Acros) following 11 days incubation (Live) or in killed controls (Killed). Peak areas are normalized to the internal standard. Note differences in y‐scale among faceted plots

With the Acros commercial NA mixture, we found compositional changes in C/Z number compared with killed controls (Figures 3 and A2). Overall, there was a 17% reduction in the intensity of 10–18 carbon compounds of the *Z* family −2 to −14, which are the major NA species in this commercial mixture. These findings showed that *P*. *fluorescens* Pf‐5 degraded specific carbon compounds in the commercial mixture of NAs. However, we also observed an increased production of compounds with larger C/Z numbers, which may be an artifact of the metabolism of endogenous substrates such as polysaccharides, lipids, polyphosphate, and DNA released from dead bacterial cells (Allesen‐Holm et al., 2006; Bradley et al., 2019; Dawes & Ribbons, 1964; Goldfine, 1972). Quesnel et al. (2011) also showed a loss of 11–17 carbon compounds of the *Z* family −2 with the unicellular alga *Dunaliella tertiolecta* with tailings associated NAs. Although *P*. *fluorescens* Pf‐5 was able to degrade *n*‐BPBA and the Acros commercial NA mixture, it should be noted that these NAs are not the same as those found in OSPW.

### 3.2 Overview of LC‐MS/MS shotgun proteomic analysis

**FIGURE 4:**
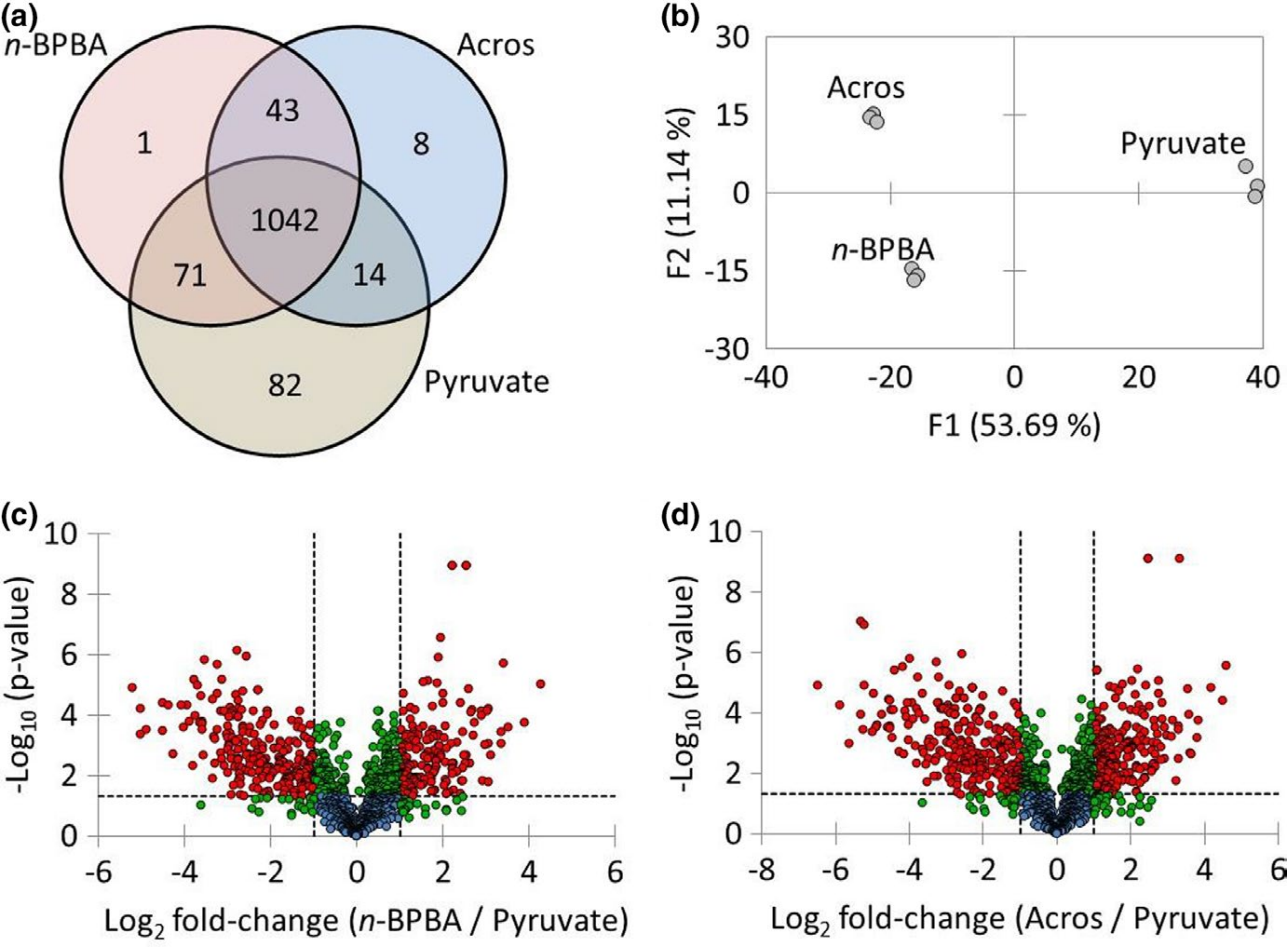
Overview of the proteomic analysis of *P*. *fluorescens* Pf‐5 growing on naphthenic acids (NAs) compared with pyruvate controls. Venn diagram comparing the common and unique proteins detected and identified during growth on the three substrates. (a) PCA analysis highlighting highly similar replicate proteomes that differ significantly with growth substrate (b), Volcano plots of normalized LC‐MS/MS spectral counts comparing *P*. *fluorescens* Pf‐5 during growth on NAs compared with pyruvate controls (c and d). Red points: proteins where *p* < 0.05 and above two‐fold difference; the green point above horizontal dotted line: proteins where *p* < 0.05 but below two‐fold difference; green points below horizontal dotted line: proteins above two‐fold difference but not statistically significant; blue points: proteins below two‐fold differential expression and not statistically significant; horizontal dashed line: *p* = 0.05; vertical dashed lines: two‐fold difference. Thus, all red points represent proteins that are above two‐fold difference (vertical dashed lines) and statistically significant (horizontal dashed line, *p* = 0.05)

The proteomes of *P*. *fluorescens* Pf‐5 were analyzed and a total of 139,921 spectral counts were obtained that were assigned to 2239 proteins. Following normalization, all very low abundance proteins (i.e., < mean of three normalized spectral counts in any treatment) were removed before statistical analysis, leaving 1261 proteins which represented 88% of the total detected normalized spectral counts. Over 72% (1042) of these proteins were detected during growth on all three growth substrates, with an additional 43 proteins that were only detected during growth on both *n*‐BPBA and Acros commercial NAs. A further one or eight proteins were only detected during growth on *n*‐BPBA or Acros commercial NAs respectively (Figure 4A). However, ordination analysis revealed that the proteomes were highly different between all three treatments (Figure 4B), also due to large changes in the relative abundance of many of the proteins detected during growth under all conditions. Proteomes from replicates were however highly similar within each treatment (Figure 4B).

**FIGURE 5:**
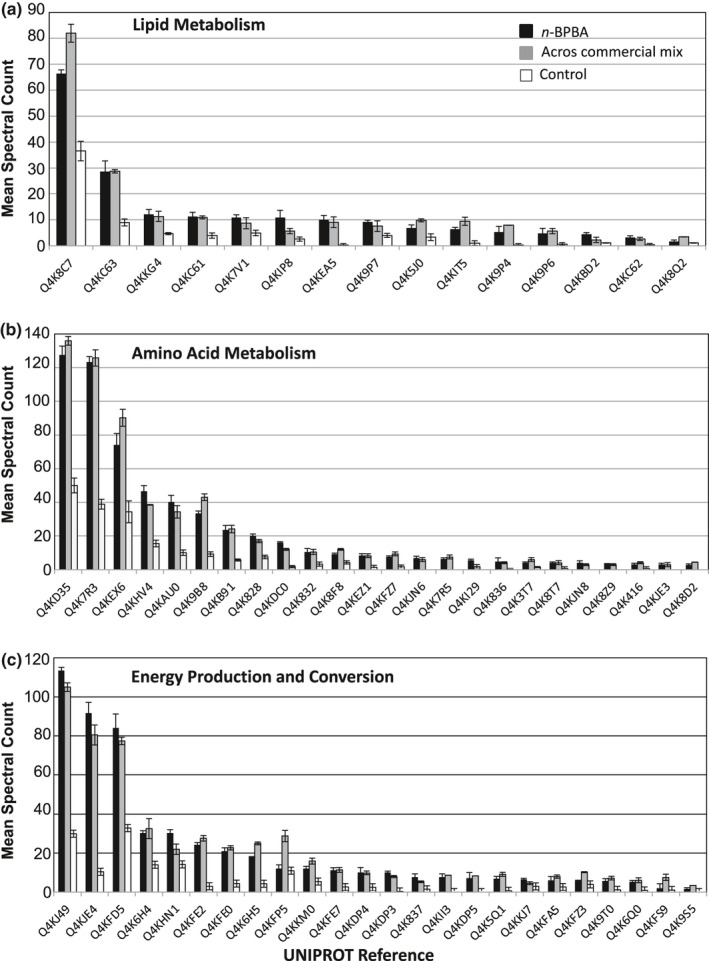
Mean spectral count *n*‐BPBA, (black bars), Acros commercial NA mixture (gray bars), and control (white bars) for metabolism functional COG categories

A total of 696 proteins were significantly differentially expressed between cells grown on *n*‐BPBA or Acros commercial NAs compared with controls. Specifically, 554 proteins were significantly differentially expressed between cells grown on *n*‐BPBA versus pyruvate (Figure 4C), of which 274 were significantly higher in relative abundance when grown on *n*‐BPBA. Similarly, 631 proteins were significantly differentially expressed with cells grown on commercial NAs (Figure 4D) of which, 314 were significantly higher in relative abundance in the cells grown on Acros commercial NAs (Table S1 at https://doi.org/10.5281/zenodo.4696143). Of the significantly differentially expressed proteins identified when *P*. *fluorescens* Pf‐5 was exposed to NAs, several were related to transport and metabolism (e.g., lipid and amino acid metabolism) as well as energy production and conversion (based on COG ontology) (Table S1 at https://doi.org/10.5281/zenodo.4696143, Figure 5).

### 3.3 Transporter proteins and outer membrane porins

Several putative membrane transporter proteins significantly increased in relative abundance with both NA treatments, including a long‐chain fatty acid transport protein (UniProt ID Q4K8C7, *p* = 0.000); an arginine–ornithine antiporter (Q4K7R5 *p* = 0.001); a transporter substrate‐binding protein (Q4K8Z9 *p* = 0.000); a sodium/ proline symporter PutP (Q4KJE3, *p* = 0.015); and a major facilitator family transporter (Q4K562, *p* = 0.048). Of particular note was a significant upregulation in putative glucose ATP (energy‐dependent efflux pumps)‐binding cassette (ABC) transporters (e.g., Q4K7T5, Q4K7T2, Q4KI80, Q4K401, all *p* < 0.03; Q4KJN6, *p* = 0.002). For example, Q4K401 increased >15‐fold, while a putative polyamine ABC transporter substrate‐binding protein (Q4KDC0, *p* = 0.00) increased 8‐fold following exposure to both NAs compared with controls. Other notable differentially expressed ABC transporter substrate‐binding proteins included Q4KFZ7 (*p* = 0.002); Q4KHV4, Q4 K828 (both *p* = 0.000) that increased between 3‐ and 5‐fold and a putative ATP‐binding cassette domain‐containing protein Q4 K832, (*p* = 0.013) which increased >2‐fold with both NA treatments.

ABC transporters are used for the uptake of many substances and it may be the observed upregulation was due to NA transport across the cell membrane. In addition, some ABC transporter proteins are important for the detoxification of xenobiotics, as they actively transport chemicals and their metabolites out of cells, protecting the cell from any potential toxic effects (Hessel et al., 2013; Klaassen & Lauren, 2010). ABC transporters have also been shown to be actively involved in the excretion of polycyclic aromatic hydrocarbons from a range of organisms (Alharbi et al., 2016; Bard, 2000). However, a variety of chemicals and metabolites can inhibit members of the ABC superfamily of transporter proteins (Kurth et al., 2015; Smital & Kurelec, 1997) including OSPW (and the potential NAs therein) (Alharbi et al., 2016). We postulate that ABC transporter inhibition occurs as a stress response following NA exposure, thus protecting the cell from NA toxic effects.

Several putative outer membrane proteins also had significantly higher spectral counts with both NA treatments compared with controls. For example, an OmpA family protein (Q4KFI8, *p* = 0.000), had >540 and >700 mean spectral counts with *n*‐BPBA and Acros commercial NAs respectively, compared to 330 spectral counts in the controls. OmpA is a proposed porin in the outer membrane of Gram‐negative bacteria such as *Pseudomonas* spp. that allows slow membrane penetration by small compounds, has been implicated in cellular responses to environmental stress (Van der Heijden et al., 2016), and plays a role in bacterial pathogenesis (Confer & Ayalew, 2013). Another outer membrane protein OprG (Q4K583, *p* = 0.000), also had significantly higher spectral counts with both NA treatments compared with controls. OprG is part of the OmpW family with proposed roles in Fe transport (McPhee et al., 2009). Regulation of Fe transport is one strategy bacteria utilize in the repair of redox stress‐induced damage (Andrews et al., 2003) and we postulate that cells were overexpressing both OmpA and OprG as a stress response to NA toxicity. Given that NAs are highly toxic to a range of organisms including bacteria (Frank et al., 2009; Morandi et al., 2015; Whitby, 2010), it was not surprising that there was a significantly higher relative abundance of transporter proteins following NA exposure (Table S1 at https://doi.org/10.5281/zenodo.4696143).

### 3.4 Lipid/fatty acid metabolism

Several proteins associated with lipid metabolism had a significantly higher relative abundance of spectral counts following NA exposure (Figure 5A). Given that alkyl side chains NAs have been found previously in Acros commercial NAs (Hao et al., 2005) and the structural similarity between alkyl‐carboxylic acid side chains and fatty acids, it is not surprising that proteins involved in lipid metabolism were overexpressed. We postulate that *P*. *fluorescens* Pf‐5 was utilizing existing fatty acid metabolism metabolic proteins (among others) during NA degradation. For example, an acyl‐CoA dehydrogenase MmgC (Q4KC62, *p* = 0.011) increased 8‐fold (with *n*‐BPBA) and 7‐fold (with Acros commercial NAs) compared with controls. This protein, along with an acyl‐CoA thioesterase II (ACOT2) (Q4K8Q2, *p* = 0.004) which itself showed >3‐fold higher expression with Acros commercial NAs, acts in the alpha‐ and beta‐oxidation of various lipids (Hunt et al., 2012) and so their overexpression is likely to be a response to the upregulation of the pathways degrading acetyl/acyl‐CoAs. In mammalian systems, when ACOT2 is upregulated, beta‐oxidation capacity increases (Fujita et al., 2011; Momose et al., 2011). It is not surprising therefore that proteins associated with alpha‐ and beta‐oxidation of lipids were overexpressed given that the aerobic transformation of NAs occurs via alpha‐/ beta‐oxidation (Blakley & Papish, 1982; Johnson et al., 2011; Rontani & Bonin, 1992). In another study, genome sequence analysis of *Cupriavidus gilardii* CR3 revealed that degradation of cyclohexane carboxylic acid (CHCA) undergoes an initial ring cleavage and that the products of which are further oxidized by beta‐oxidation via several pathways including a mechanism similar to that used for fatty acid oxidation (Wang et al., 2015). The peripheral ring‐cleavage process in *C*. *gilardii* CR3 observed by Wang et al. (2015), was similar to the proposed process for aerobic oxidation of cyclohexane carboxylic acid by aerobic oxidation pathways in *Pseudomonas putida* (Blakley & Papish, 1982). In another study, the gene cluster *chcpca* was transcriptionally induced during the biodegradation of the alicyclic model NA compounds CHCA and cyclopentanecarboxylic acid (CPCA) by *Rhodococcus aetherivorans* BCP1 and has been proposed to be involved in the beta‐oxidation pathway (Presentato et al., 2018).

In addition to ACOT, an enoyl‐CoA hydratase (Q4KC63, *p* = 0.001) was also detected in significantly more abundance, increasing by 3‐fold for both NA treatments compared to controls (Figure 5A). Enoyl‐CoA hydratase is essential for metabolizing fatty acids in the beta‐oxidation pathway to produce acetyl‐CoA and ATP. A putative enoyl‐CoA hydratase was recently shown to contribute to biofilm formation in an antibiotic tolerant denitrifying bacterium and pathogen *Achromobacter xylosoxidans* (Cameron et al., 2019). When the gene (*echA*) encoding a putative enoyl‐CoA hydratase was disrupted, a decrease in biofilm accumulation occurred, increasing the organism's susceptibility to antibiotics (Cameron et al., 2019). In our study, a poly‐beta‐1,6‐N‐acetyl‐D‐glucosamine N‐deacetylase protein (Q4KKC4, *p* = 0.000), which is involved in biofilm formation (Wang et al., 2004), was also significantly more abundant when cells were exposed to both NA treatments, remaining undetected in the control. Although biofilm formation was not measured herein, it is well known that biofilms facilitate substrate degradation (Chakraborty et al., 2012; Nicolella et al., 2000; Singh et al., 2006) including NAs (Choi et al., 2014; Demeter et al., 2015; Folwell et al., 2016; Golby et al., 2012), and overexpression of these proteins involved in biofilm formation may facilitate NA removal in OSPW.

Several putative dehydrogenases had significantly higher relative abundance with both NA treatments (Figure 5A), including an acyl‐CoA dehydrogenase (Q4K5J0, *p* = 0.005); 3‐hydroxyisobutyrate dehydrogenase (Q4KIP8, *p*=0.016); a dihydrolipoyl dehydrogenase (Q4KDP5, *p* = 0.039) a Glu/Leu/Phe/Val dehydrogenase (Q4KI29, *p* = 0.003); and an isovaleryl‐CoA dehydrogenase (Q4K9P7, *p* = 0.026) which participates in valine, leucine, and isoleucine degradation. In addition, several putative oxidoreductases, carboxylases and transferases increased significantly in relative abundance with both NA treatments including FAD‐dependent oxidoreductase (Q4KED0, *p* = 0.026); NADH quinone oxidoreductase (Q4K9S5, *p* = 0.012); NAD(P)‐dependent oxidoreductases (Q4KBD2, Q4KC60, *p* < 0.014); FAD‐binding oxidoreductase (Q4KEZ1, *p* = 0.003); flavin oxidoreductase/NADH oxidase (Q4KHN1, *p* = 0.001); NADH quinone oxidoreductases (Q4K9T0, Q4K9S5, *p* < 0.012); the acetyl/propionyl/methylcrotonyl‐CoA carboxylases (Q4K9P4, Q4K9P6, *p* < 0.024); acetyl‐CoA C‐acetyltransferase (Q4KC61, *p* = 0.003); and acetyl‐CoA C‐acetyl transferases (Q4KIT5, Q4KEA5, *p* < 0.003). Additionally, a flavoprotein (Q4KFP5, *p* = 0.001) and long‐chain fatty acid‐CoA ligase (Q4K7V1, *p* = 0.029) increased significantly in relative abundance with both NA treatments compared to controls.

It was notable that five proteins were significantly differentially expressed with the Acros commercial NAs but not detected with either *n*‐BPBA or the controls. These were three putative dehydrogenases (acyl‐CoA dehydrogenases (Q4K8Z4, Q4KFF6, *p* < 0.005); 3‐hydroxyacyl‐CoA dehydrogenase (Q4KFM7, *p* = 0.000); and two carboxylases, namely acetyl/ propionyl/ methylcrotonyl‐CoA carboxylase alpha subunit (Q4K8Z2, *p* = 0.000) and acyl‐CoA carboxylase beta subunit (Q4K8Z5, *p* = 0.000) (Table S1 at https://doi.org/10.5281/zenodo.4696143). Acetyl‐CoA carboxylase catalyzes the carboxylation of acetyl‐CoA to produce malonyl‐CoA, which is a substrate for fatty acid biosynthesis (Tong, 2005). It is possible that this suite of proteins was involved in degrading certain carboxylic acids within the Acros commercial mixture, possibly the 10–18 carbon compounds of the *Z* family −2 to −14 that we observed a reduction in (Figure 3), although this is yet to be confirmed.

**FIGURE 6:**
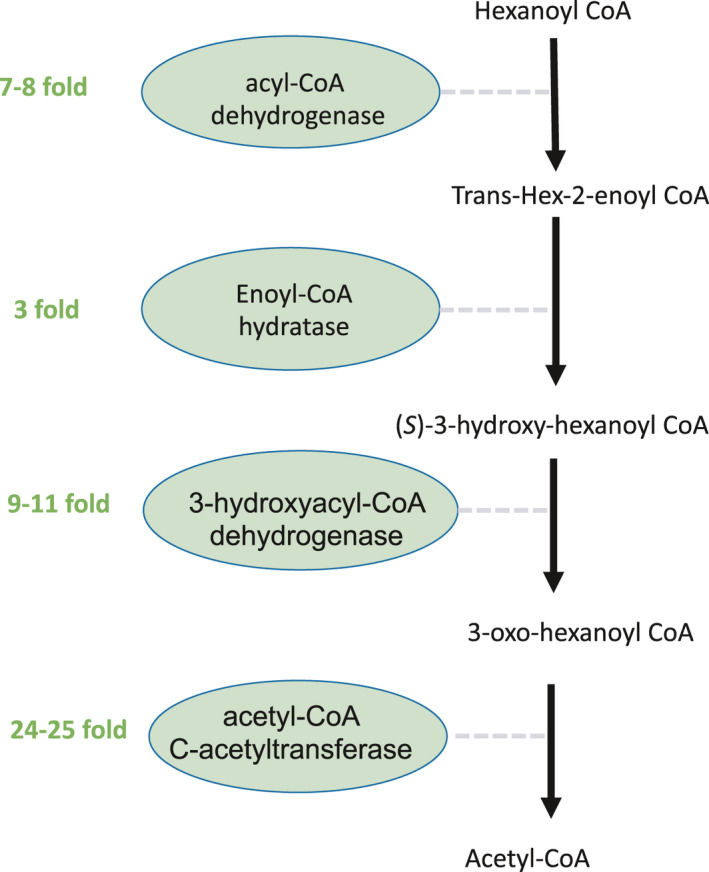
Partial enzymatic pathway diagram for fatty acid degradation in *Pseudomonas fluorescens* Pf‐5

**FIGURE A3:**
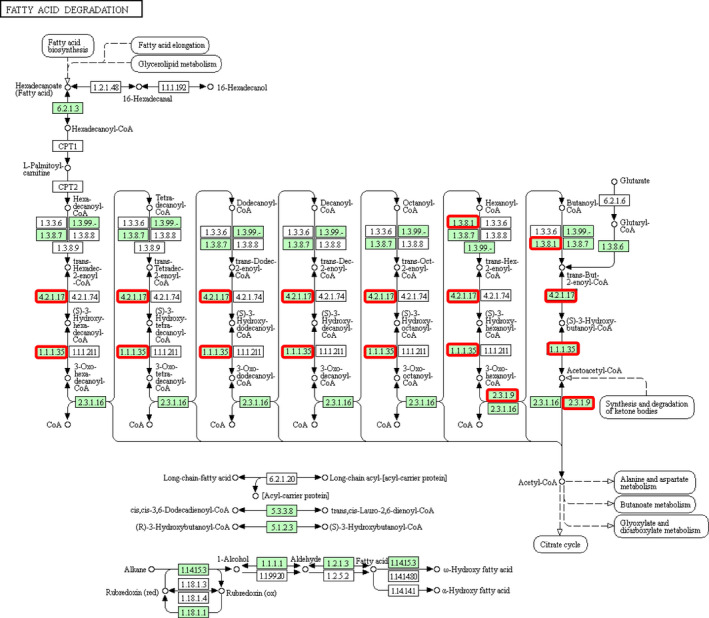
KEGG pathway diagram for fatty acid degradation in Pseudomonas fluorescens Pf‐5. Enzymes 1.3.8.1 (acyl‐CoA dehydrogenase, Q4KC62) and 2.3.1.9 (acetyl‐CoA C‐acetyltransferase, Q4KEA5) were upregulated by 27 to 30‐fold and 7 to 9‐fold on the different NA mixtures. Enzymes 1.1.1.35 (3‐hydroxyacyl‐CoA dehydrogenase, Q4KC60) and 4.2.1.17 (enoyl‐CoA hydratase, Q4KC63) were upregulated by between 3 to 4‐fold and 3.5‐fold, respectively. Upregulated enzymes are highlighted in red boxes

Importantly, we found multiple proteins involved in sequential reactions in fatty acid degradation were upregulated with both NA treatments (Figure A3), and an example of such a pathway is given (Figure 6). We postulate that *P*. *fluorescens* Pf‐5 cells were utilizing their existing fatty acid metabolism machinery to metabolize the NAs tested. Since these fatty acid pathways are conserved, a wide variety of species are likely to have the enzymatic potential to biodegrade NAs and a range of NA‐degrading microorganisms have been identified (reviewed in Skeels & Whitby, 2019). It is possible, however, that under certain conditions Pseudomonads may have a competitive advantage, not only by withstanding NA toxicity but as likely NA‐degrading genera. For example, Johnson et al. (2011) showed that Pseudomonads increased in abundance during the degradation of certain NAs in mixed communities. This is supported by Folwell et al. (2020) who showed that enrichment cultures dominated by Pseudomonads, especially *P*. *stutzeri*, degraded diamondoid carboxylic acids (e.g., adamantane‐1‐carboxylic acid (A1CA)), whereas other organisms such as *Rhodococcus sp*. could not degrade tricyclic adamantane carboxylic acid, despite being able to degrade aliphatic and alicyclic carboxylic acid compounds (Presentato et al., 2018). Thus, targeting Pseudomonads in OSPW may be a bioremediation strategy for oil sands operators for enhanced NA removal.

### 3.5 Amino ​acid metabolism

**FIGURE A4:**
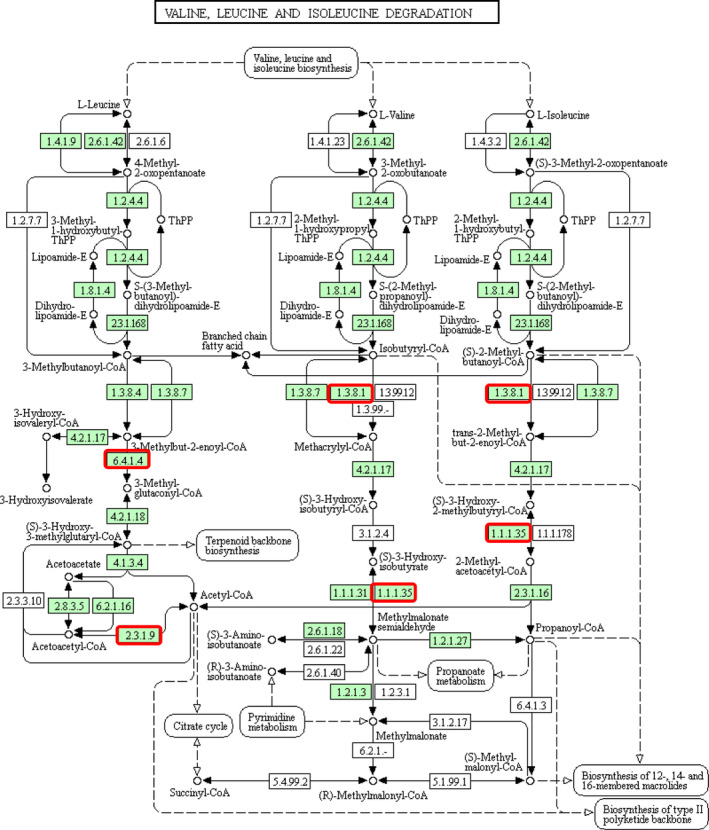
KEGG pathway diagram for amino acid degradation in Pseudomonas fluorescens Pf‐5. Upregulated enzymes 1.3.8.1 (acyl‐CoA dehydrogenase, Q4KC62) and 6.4.1.4 (acetyl/propionyl/methylcrotonyl‐CoA carboxylase subunit alpha, Q4K9P4) are highlighted in red boxes. Enzyme 1.1.1.35 (3‐hydroxyacyl‐CoA dehydrogenase, Q4KC60) and enzyme 2.3.1.9 (acetyl‐CoA C‐acetyltransferase, Q4KEA5) are also shown

A total of 24 proteins significantly upregulated by >2‐fold were related to amino acid metabolism (Figure 5B) and notable upregulated proteins are detailed in the amino acid metabolism KEGG pathway (Figure A4). Specifically, an ornithine carbamoyltransferase (OTCase, Q4K7R3, *p* = 0.000) was upregulated 3‐fold (with both *n*‐BPBA and Acros commercial NAs) compared with controls. There are two classes of OTCase, anabolic and catabolic that function in arginine metabolism (Cunin et al., 1986). All anabolic OTCases, except those found in *Pseudomonas* spp. catalyze both directions of the reaction (Cunin et al., 1986). In some prokaryotes including ​Pseudomonads, OTCase can degrade arginine by the reverse mechanism in the arginine dihydrolase pathway (Cunin et al., 1986). In *P*. *putida*, OTCase is inhibited by relatively high concentrations of arginine (Stalon et al., 1977).

In addition to OTCase, another protein that had significantly higher abundance with both NA treatments was a putative glutaminase‐asparaginase AnsB (Q4KEX6, *p* = 0.001), which is widely distributed in microorganisms and had a wide substrate specificity (Hüser et al., 1999; Wriston & Yellin, 1973). Another protein that increased in relative abundance (between 11 to 12‐fold) with both NA treatments was a putative arginine N‐succinyl transferase (Q4 K836, *p* = 0.039) which uses succinyl‐CoA and L‐arginine as substrates to produce CoA and N_2_‐succinyl‐L‐arginine. Other proteins that significantly increased >2‐fold following exposure to both NA treatments were: NAD glutamate dehydrogenase (Q4KD35, *p* = 0.000), which is found in most microbes and converts glutamate to α‐ketoglutarate (Wootton, 1983); D‐amino acid dehydrogenase (Q4K3T7, *p* = 0.007), 3‐deoxy‐7‐phosphoheptulonate synthase class II (Q4K8T7, *p* = 0.028), glycine dehydrogenase aminomethyl‐transferring protein (Q4K416, *p* = 0.018); urocanate hydratase (Q4KJN8, *p* = 0.010) involved in histidine degradation; a Fe(II)‐containing non‐heme oxygenase (4‐hydroxyphenylpyruvate dioxygenase, Q4 KB91, *p* = 0.001) involved in tyrosine catabolism, a dipeptidase (Q4KAU0, *p* = 0.001) and an aminopeptidase (Q4K8F8, *p* = 0.001) (Figure 5B).

### 3.6 Energy production and conversion, secondary metabolism

Several proteins putatively involved in secondary metabolism also had a higher relative abundance with both NA treatments (Figure 5C) including a glycerophosphodiester phosphodiesterase (Q4KFA5, *p* = 0.026); alcohol dehydrogenases (e.g., Q4K4Z2, Q4K9B8, both *p* < 0.019) which catalyzes the conversion of the primary alcohol to an aldehyde; and aldehyde dehydrogenases which oxidize aldehydes to carboxylic acids (e.g., Q4KAB3, *p* = 0.025, Q4K837, *p* = 0.011) (Table S1, Figure 5C). It is notable that aldehyde dehydrogenases also catalyze xenobiotic metabolism (Sladek, 2002) and could potentially be involved in NA metabolism herein, although this remains inconclusive.

One protein identified as a putative alpha‐ketoacid dehydrogenase subunit (Q4KDP3, *p* = 0.000), which catalyzes the oxidative decarboxylation of branched, short‐chain alpha‐ketoacids significantly increased 25‐fold (with *n*‐BPBA) and 21‐fold (with Acros commercial NAs). Other notable proteins were: proline dehydrogenase (Q4KJE4, *p* = 0.000); aconitate hydratase (Q4KFD5, *p* = 0.001); and succinate dehydrogenase (Q4KFZ3, *p* = 0.001) which converts succinate to fumarate as part of the Krebs cycle. Several Cytochrome b, c, or d family proteins including multiple subunits of cytochrome c oxidases including Q4KFE2, Q4K6H5, Q4KFE7, Q4KKM0, Q4KFE0, Q4KKJ7, Q4K6H4, and Q4K5Q1, (all *p* < 0.004) and Q4K6Q0 (*p* = 0.01), along with an iron–sulfur cluster‐binding protein (Q4KII3, *p* = 0.002), which are all involved in the electron transport chain also had significantly higher abundance with both NA treatments compared with controls.

Several proteins relating to inorganic ion metabolism were also significantly differentially expressed with both NA treatments. For example, a copper‐containing nitrite reductase (CuNiR) which reversibly reduces nitrite to NO (Q4K5B4, *p* = 0.000) increased >8 to 9‐fold. Increased expression of this denitrifying enzyme could arise from the ammonium in the MSM medium or from the liberation of nitrite from nitrate esters. In support of this, a putative pentaerythritol trinitrate reductase (Q4KHN1, *p* = 0.001) which reductively liberates nitrite from nitrate esters and degrades xenobiotics such as 2,4,6‐trinitrotoluene (TNT) (French et al., 1996, 1998) was significantly increased with both NA treatments. Ammonium oxidation pathways have been well characterized in Pseudomonads and typically involve ammonia monooxygenase producing hydroxylamine which is subsequently oxidized to nitrite (Hollocher et al., 1981). As a result, CuNiR could then be upregulated to deal with excess nitrite. An azurin‐related protein (Q4 KJ49, *p* = 0.000) also had high spectral counts with both NA treatments. Azurin is a periplasmic Cu‐containing cupredoxin protein capable of electron transfer reactions including being an electron donor to CuNiR in the denitrification pathway (Zumft, 1997). It has also been implicated as a part of cellular response to redox stress. Notably, azurin knockout strains of *P*. *aeruginosa* showed increased sensitivity to hydrogen peroxide or paraquat redox stress but with no impairment to growth on nitrite or nitrite (Vijgenboom et al., 1997).

Molybdenum is an essential micronutrient for microorganisms and molybdoenzymes are widespread among prokaryotes where they catalyze steps in carbon, sulfur, and nitrogen metabolism (Mendel, 2013; Mendel & Schwarz, 2011; Schwarz et al., 2009), are involved in chemotaxis toward electron acceptors, environmental stress responses (Baraquet et al., 2009; Leimkuhler & Lobbi‐Nivol, 2016; Schwartz & Mendel, 2006), and play a role in pollutant detoxification (e.g., chromium Cr(VI) and arsenic (III)) (Chovanec et al., 2012; Islam et al., 2004; Kruger et al., 2013; Slyemi & Bonnefoy, 2012). When competing anions are present, molybdoenzymes require specific uptake systems including high‐affinity ABC transporters (Hagen, 2011) and this supports the upregulation of ABC transporters found herein. In our study, a molybdenum (Moco) cofactor biosynthesis protein (Q4KAB0, *p* = 0.035) was significantly differentially expressed >11‐fold (with Acros commercial NAs) and 3.5‐fold (with *n*‐BPBA). Yet interestingly, membrane nitrate reductase (Nar) (among other proteins known to contain a Moco cofactor in *P*. *fluorescens* Pf‐5) was not found to be upregulated in our study. Molybdenum metabolism is tightly connected to Fe‐S cluster synthesis (Mendel, 2013) and in our study, bacterioferritin (*Bfr2*), an iron uptake protein (Q4 K560, *p* = 0.000) with likely roles in iron storage, mobilization, and homeostasis (Rivera, 2017), was also significantly expressed (between 5 and 7.5‐fold with both NA treatments). In *P*. *aeruginosa*, *Bfr2* bacterioferritin provides resistance to hydrogen peroxidase (Ma et al., 1999) and we postulate that *Bfr2* (along with molybdoenzyme) upregulation was an oxidative stress response to NA toxicity and both were involved in NA detoxification, although this remains to be determined.

### 3.7 Other cellular functions: cell wall biogenesis and chemotaxis

Proteins associated with other cellular functions were also significantly more abundant (by >2‐fold) in both NA treatments compared with controls included a signal transductor histidine kinase (Q4K8D2, *p* = 0.000); a polysaccharide export protein (Q4K8Y1, *p* = 0.012) which is involved in cell wall biosynthesis (Table S1 at https://doi.org/10.5281/zenodo.4696143) and several methyl‐accepting chemotaxis proteins (e.g., Q4KE19, Q4KBC3, <*p* = 0.003) which are involved in cell motility were also in higher relative abundance in both the NA treatments and are likely a chemotactic response to NA toxicity.

## 4 CONCLUSIONS

Little was previously known about the mechanisms involved in NA biodegradation and the enzymes or other proteins involved in the metabolism of this class of OSPW contaminants. Here we sought to elucidate proteins significantly upregulated during NA biodegradation of a model NA and commercial NA mixture with *P*. *fluorescens* Pf‐5 as a model microorganism. Several differentially expressed proteins were identified following NA exposure that were involved in metabolism—lipid, fatty acid, and amino acid degradation pathways—suggesting that *P*. *fluorescens* Pf‐5 may be utilizing its existing lipid, fatty acid metabolism metabolic proteins (among others) during NA biodegradation. Multiple membrane porins, transporters, and chemotaxis proteins were also significantly upregulated and likely represent a general cellular response to oxidative stress and the cell's detoxification mechanisms to protect the cell from environmental stress and NA toxicity. Additional proteins were also highly upregulated, such as CuNiR and Moco biosynthesis proteins, though their significance in NA degradation remains to be determined. In conclusion, we provide new empirical evidence of potential proteins that could be targeted in further overexpression or synthetic biology studies with more authentic OSPW NAs as a novel approach for improving OSPW reclamation in the future.

## CONFLICT OF INTEREST

None declared.

## AUTHOR CONTRIBUTIONS

**Boyd A. McKew:** Data curation (supporting); Formal analysis (supporting); Investigation (supporting); Methodology (supporting); Validation (supporting); Visualization (supporting); Writing‐original draft (supporting). **Richard Johnson:** Conceptualization (supporting); Data curation (supporting); Formal analysis (supporting); Investigation (supporting); Methodology (supporting); Validation (supporting); Writing‐original draft (supporting). **Lindsey Clothier:** Methodology (supporting); Validation (supporting); Writing‐original draft (supporting). **Karl Skeels:** Formal analysis (supporting); Investigation (supporting); Methodology (supporting); Writing‐original draft (supporting). **Matthew S. Ross:** Conceptualization (supporting); Data curation (supporting); Formal analysis (supporting); Investigation (supporting); Methodology (supporting); Validation (supporting); Writing‐original draft (supporting). **Metodi Metodiev:** Methodology (supporting); Resources (supporting); Writing‐original draft (supporting). **Max Frenzel:** Methodology (supporting); Supervision (supporting); Visualization (supporting); Writing‐original draft (supporting). **Lisa M. Gieg:** Investigation (supporting); Methodology (supporting); Validation (supporting); Writing‐original draft (supporting). **Jonathan W. Martin:** Investigation (supporting); Methodology (supporting); Validation (supporting); Writing‐original draft (supporting). **Michael A. Hough:** Conceptualization (supporting); Data curation (supporting); Formal analysis (supporting); Funding acquisition (equal); Investigation (supporting); Methodology (supporting); Project administration (supporting); Resources (equal); Software (lead); Supervision (equal); Validation (supporting); Writing‐original draft (supporting). **Corinne Whitby:** Conceptualization (lead); Funding acquisition (lead); Investigation (lead); Methodology (supporting); Project administration (lead); Resources (lead); Supervision (lead); Validation (lead); Writing‐original draft (lead).

## ETHICS STATEMENT

None required.

## Data Availability

All data are provided in full in this paper except Table S1 (Full proteomics dataset) which is available in the Zenodo repository at https://doi.org/10.5281/zenodo.4696143
